# Optimization of Heterotrophic Culture Conditions for the Microalgae *Euglena gracilis* to Produce Proteins

**DOI:** 10.3390/md21100519

**Published:** 2023-09-29

**Authors:** Weiying Xie, Xiaojie Li, Huo Xu, Feng Chen, Ka-Wing Cheng, Hongbin Liu, Bin Liu

**Affiliations:** 1 SZU-HKUST Joint Ph.D. Program in Marine Environmental Science, Shenzhen University, Shenzhen 518060, Chinahxuay@connect.ust.hk (H.X.); 2 Shenzhen Key Laboratory of Food Nutrition and Health, Shenzhen Key Laboratory of Marine Microbiome Engineering, Institute for Advanced Study, Shenzhen University, Shenzhen 518060, China; 3 Institute for Innovative Development of Food Industry, Shenzhen University, Shenzhen518060, China; 4 Department of Ocean Science, The Hong Kong University of Science and Technology, Hong Kong SAR, China; 5 Hong Kong Branch of Southern Marine Science & Engineering Guangdong Laboratory, The Hong Kong University of Science and Technology, Hong Kong SAR, China

**Keywords:** dietary protein, *Euglena gracilis*, heterotrophic, nitrogen source, C/N ratio, amino acid profile

## Abstract

*Euglena gracilis* is one of the few permitted edible microalgae. Considering consumer acceptance, *E. gracilis* grown heterotrophically with yellow appearances have wider food industrial applications such as producing meat analogs than green cells. However, there is much room to improve the protein content of heterotrophic culture cells. In this study, the effects of nitrogen sources, temperature, initial pH, and C/N ratios on the protein production of *E. gracilis* were evaluated under heterotrophic cultivation. These results indicated that ammonium sulfate was the optimal nitrogen source for protein production. The protein content of *E. gracilis* cultured by ammonium sulfate increased by 113% and 44.7% compared with that cultured by yeast extract and monosodium glutamate, respectively. The manipulation of the low C/N ratio further improved *E. gracilis* protein content to 66.10% (*w*/*w*), which was 1.6-fold of that in the C/N = 25 group. Additionally, amino acid analysis revealed that the nitrogen-to-protein conversion factor (NTP) could be affected by nitrogen sources. A superior essential amino acid index (EAAI) of 1.62 and a balanced amino acid profile further confirmed the high nutritional value of *E. gracilis* protein fed by ammonium sulfate. This study highlighted the vast potency of heterotrophic cultured *E. gracilis* as an alternative dietary protein source.

## 1. Introduction

The global shortage of protein supply will be one of the biggest problems in the next 30 years, especially when the population is projected to exceed 9.1 billion, and the meat demand might rise to 455 million tons by 2050 [1,2]. Recently, animal protein production was considered unsustainable because traditional animal agriculture is harmful to the environment, requiring large amounts of resources (e.g., land and water) and emitting many greenhouse gases [1,2]. Therefore, the animal-free protein produced by legumes, cereals, and microbial species is increasingly popular and has shown increasing market share in recent years [3].

Microalgae have emerged as a promising bio-economy practical resource for plant-based protein production with the advantages of high protein content (up to 71%), less land requirement, no season limitation, and a higher growth rate than traditional crops [4,5]. *Euglena gracilis* has several advantages to be applied as a single-cell protein resource among various microalgal species. For example, *E. gracilis* was approved as a dietary resource in Europe, and was also permitted to be used in China without the daily intake limitation [6]. Meanwhile, it contains 18 amino acids with a high proportion of essential sulfur amino acids, providing a healthy balance of essential amino acids to meet daily protein intake requirements [7,8]. In addition, *E. gracilis* is highly digestible as it lacks a rigid celluloid cell wall [8]. More importantly, *E. gracilis* can produce yellow-color biomass during their heterotrophic growth due to the degradation of secondary-endosymbiosis chloroplast [9], giving it wider application in the food industry such as meat analog [6].

The protein content of *E. gracilis* can reach up to 60% (*w*/*w*) and 47% (*w*/*w*) under mixotrophic and autotrophic culture conditions, respectively [10,11]. However, *E. gracilis* grown heterotrophic mainly accumulates storage carbohydrates (paramylon), and the protein content could be less than 20% (*w*/*w*) [12]. The low protein production of *E. gracilis* under heterotrophic conditions limits its wide application as a protein resource.

Nitrogen sources were one of the essential factors for microalgae protein biosynthesis [13]. *Chlorella sorokiniana* prefers ammonium salt for protein production [14], while *Scenedesmus obliquus* prefers urea [15]. The prevailing view is that sodium glutamate and ammonium salt can be utilized by *E. gracilis* [10,16], while nitrate and urea are not suitable substrates due to the lack of nitrate reductase and urease *in E. gracilis* [17]. Nitrogen starvation is a commonly used strategy to promote lipid accumulation in microalgae, while a decrease in the C/N ratio was also found to be beneficial for protein accumulation by various microalgae such as *C. sorokiniana* [14], *Porphyridium purpureum* [18], and *Dunaliella* sp. [19]. However, Regnault et al. (1990) claimed that the C/N ratio had little effect on protein content in *E. gracilis* [20].

It is necessary to systematically explore the optimal cultivation strategies for *E. gracilis* under heterotrophic conditions for improving its protein content. The influences of the nitrogen source and C/N ratio on protein accumulation in *E. gracilis* are not clearly studied. Meanwhile, initial pH and temperature have not yet been explored its effect on protein production in this species as well. In the present study, it is hypothesized that *E. gracilis* growing heterotrophically could have a protein content improvement by optimizing its cultivation conditions. The effects of the nitrogen source, temperature, initial pH, and C/N ratio on the protein content and protein yield of *E. gracilis* under heterotrophic conditions were determined. Other main cell compositions were also analyzed to further find the flux direction of the carbon skeleton under different cultivation conditions. The amino acid profile analysis was also conducted to evaluate its protein nutritional quality.

## 2. Results and Discussion

### 2.1. The Effects of Nitrogen Sources on the Protein Content and Protein Yield

**Figure 1:**
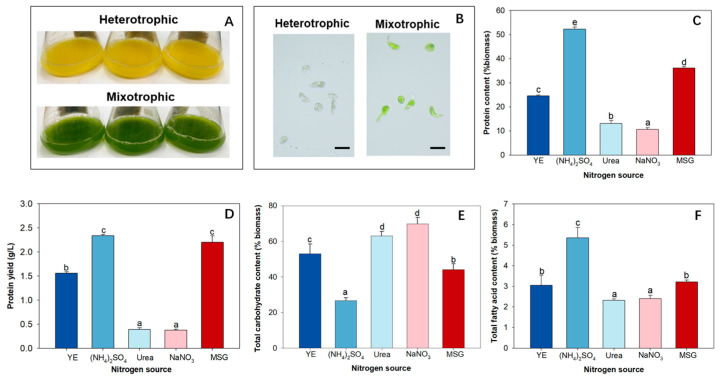
(**A**) *Euglena gracilis* cultured under heterotrophic and mixotrophic conditions. (**B**) Light micrographs of *E. gracilis* under heterotrophic and mixotrophic conditions (Bars: 50 μm). (**C**) Protein content, (**D**) protein yield, (**E**) total fatty acid content, and (**F**) total carbohydrate content of *E. gracilis* cultured with different nitrogen sources under 32 °C/C/N = 17/initial pH 3.5. (YE: yeast extract, MSG: monosodium glutamate); bars followed by the same letter are not significantly different (*p* < 0.05).

As shown in Figure 1A,B, *E. gracilis* exhibited a yellow appearance under heterotrophic cultivation, while it was presented as green under the mixotrophic culture. The heterotrophic cultures only contained 0.051 ± 0.001 mg/g chlorophyll a and 0.019 ± 0.001mg/g chlorophyll b, much less than that of mixotrophic cultures, which contained 7.895 ± 0.381 mg/g chlorophyll a and 2.983 ± 0.110 mg/g chlorophyll b (Figure S1).

Five commonly used nitrogen sources were evaluated for protein production by *E. gracilis.* Like other microalgae, *E. gracilis* showed a disparity of protein content among different nitrogen sources (Figure 1C). *E. gracilis* cultured with ammonium sulfate showed the highest protein content of 52.26 ± 0.91%(*w*/*w*), which was significantly higher than that of the yeast extract group (24.53 ± 0.37%) and monosodium glutamate (MSG) group (36.11 ± 0.63%) (Figure 1C). In contrast, cells supplemented with urea and sodium nitrate not only had lower protein contents (<15%) but also lower protein yields (0.39 ± 0.03 g/L and 0.38 ± 0.02 g/L, respectively) (Figure 1D). This implied that nitrate and urea could not be assimilated effectively by *E. gracilis* to produce protein [21].

The total fatty acid content displayed a similar trend to the protein content across the different nitrogen source treatments (Figure 1E). Interestingly, the sodium nitrate (69.86 ± 3.60%) and urea (63.15 ± 2.51%) groups generated significantly higher total carbohydrate content than other groups (Figure 1F), in contrast to the protein content trend observed. Considering the low content of total fatty acid across the treatments, our results implied that the metabolic conversion between protein and carbohydrate was the main pathway in *E. gracilis* cultivated by different nitrogen sources. Overall, the nitrogen screening experiment highlighted that ammonium sulfate was the potential optimal nitrogen source for protein biosynthesis, which yielded cells with a protein content of up to 52.26% (*w*/*w*).

The substantial variation in protein content among different nitrogen sources aroused our interest to further investigate the nitrogen assimilation pathway in *E. gracilis*. However, due to the high metabolic variability caused by the complex nature of yeast extract and the ineffectiveof *E. gracilis* to utilize urea and sodium nitrate, the nitrogen source metabolism of ammonium sulfate and MSG are mainly discussed.

It is widely recognized that ammonium assimilation is primarily related to the irreversible GS/GOGAT pathway and reversible GDH pathway (Figure S2) [22]. The GDH pathway is considered a shunt in the whole ammonium assimilation pathway that could divert the carbon skeleton from nitrogen metabolism to carbon metabolism via the TCA cycle [23]. Thus, it is speculated that the observed disparity in protein synthesis between ammonium sulfate and MSG groups may be attributed to the feedback inhibition as the following indications: (1) When using ammonium sulfate as a nitrogen source, ammonium is preferentially consumed along with 2-OG as a carbon skeleton via the nitrogen assimilation pathway and finally produce glutamic acid for further producing other amino acids as protein building blocks [22]. (2) Since glutamic acid is the end-product of GDH, the appearance of a large amount of glutamate might conversely be catalyzed by GDH to form ammonium ions and 2-OG. However, 2-OG could be utilized as a carbon skeleton to produce carbohydrates via the TCA cycle. The feedback inhibition of glutamate may lead to the loss of carbon skeleton in the nitrogen assimilation procedure, resulting in a decrease in protein production in the MSG group compared to the ammonium sulfate group. Further investigation is needed to fully elucidate the mechanism underlying the protein content disparity caused by different nitrogen sources.

### 2.2. The Effects of Nitrogen Sources on the Amino Acid Profile

**Figure 2:**
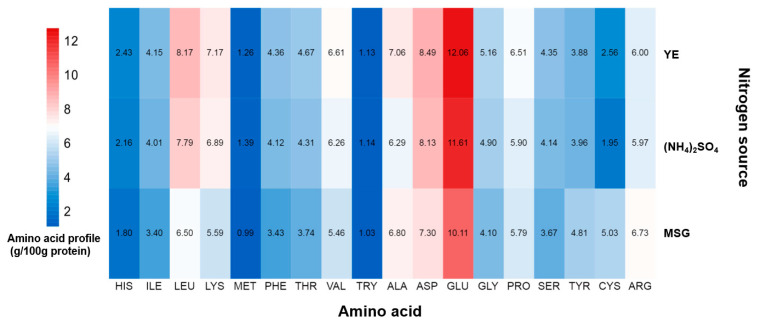
Heatmap of amino acid distribution (g/100 g protein) of *E. gracilis* cultured with different nitrogen sources under heterotrophic cultivation conditions. (HIS: histidine, ILE: isoleucine, LEU: leucine, LYS: lysine, MET: methionine, PHE: phenylalanine, THR: threonine, VAL: valine, TRY: tryptophan, ALA: alanine, ASP: aspartic acid, GLU: glutamic acid, GLY: glycine, PRO: proline, SER: serine, TYR: tyrosine, CYS: cysteine, ARG: arginine) (YE: yeast extract, MSG: monosodium glutamate) The number showed in heatmap is the average content of each amino acid.

To evaluate the nutritional value and NTP of *E. gracilis* protein, the amino acid profiles under different nitrogen sources were determined. The samples fed with urea and sodium nitrate were not analyzed since these two nitrogen sources cannot be utilized by *E. gracilis*. As shown in Figure 2, *E. gracilis* protein had a balanced amino acids profile, including cysteine and methionine, which are typically insufficient in legumes, and lysine, which is the first limiting amino acid in cereals [2,24]. Furthermore, glutamic acid, aspartic acid, and leucine were three major amino acids in *E. gracilis* protein. Glutamic acid served as a precursor of many amino acid anabolisms, such as transamination to form aspartic acid and alanine [25,26]. This feature was consistent with the amino acid profile of six other microalgae species, including *Chlorella vulgaris*, *Dunaliella bardawil*, *S. obliquus*, *Arthrospira maxima*, *Spirulina platensis*, and *Aphanizomennon* sp. [27]. The MSG group contained lower level of isoleucine (3.40 ± 0.13 g/100 g protein), leucine (6.50 ± 0.27 g/100 g protein), lysine (5.59 ± 0.23 g/100 g protein), phenylalanine (3.43 ± 0.13 g/100 g protein), threonine (3.74 ± 0.16 g/100 g protein), valine (5.46 ± 0.24 g/100 g protein), tryptophan (1.03 ± 0.02 g/100 g protein), glutamic acid (10.11 ± 0.41 g/100 g protein), and glycine (4.10 ± 0.16 g/100 g protein) than that of other two groups, but higher levels of tyrosine (4.81 ± 0.33 g/100 g protein) and cysteine (5.03 ± 0.39 g/100 g protein). The amino acid profiles were similar between the yeast extract and the ammonium sulfate group. As shown in Table 1, the ammonium sulfate group contained the highest amino acid content (47.53 ± 2.08 g/100 g sample), while the yeast extract group contained the lowest amino acid content (23.53 ± 1.24 g/100 g sample). This result corresponded to the result of protein content (Figure 1C). The proportion of total essential amino acid to total amino acid (E/T) was found to be about 42% for both the yeast extract group and the ammonium sulfate group, which were comparable to that reported for *C. vulgaris* protein [25]. However, the E/T value was lower in the MSG group (37.02 ± 0.59%). The lower–E/T value suggested that MSG was not an ideal nitrogen source for protein production compared with ammonium sulfate and yeast extract, considering its nutritional value.

Based on the amino acid profile, NTP was calculated since the conventional approach of using a versatile NTP value of 6.25 may lead to an overestimation of the protein content as the presence of non-protein nitrogen compounds, such as inorganic nitrogen, nucleic acids, and amino sugars [28]. The *k_A_* values are similar among these three groups, ranging from 6.11 ± 0.01 to 6.17 ± 0. A previous study investigated the *k_P_* value of 10 different microalgal species, which ranged from 3.60 ± 0.27 to 4.99 ± 0.64 [28]. The *k_P_* and *NTP* value of *E. gracilis* sample fed with ammonium sulfate were 4.87 ± 0.13 and 5.52 ± 0.06, respectively. The *k_P_* of *E. gracilis* protein was comparable with most of the reported species but were shown to be higher than *Skeletonema costatum* (3.82 ± 0.33), *Prorocentrum minimum* (3.88 ± 0.68), and *Dunaliella tertiolecta* (3.99 ± 0.48) [28]. Recently, the *NTP* value of four industrial-applied microalgae (*Scenedesmus* sp., *C. vulgaris*, *Phaeodactylum tricornutum*, and *Nannochloropsis* sp.) were recalculated. The results showed that the *NTP* (ranging from 4.68 to 5.35) and *k_P_* (ranging from 3.00 to 4.43) values of these four microalgae were all lower than those obtained from the present study [29]. This indicated that *E. gracilis* has a lower non-protein–nitrogen content than microalgae species [29]. These findings suggested that heterotrophic cultivated *E. gracilis*, when fed with ammonium sulfate, could be considered a reliable and promising protein source.

### 2.3. The Effect of Initial pH on Protein Content and Protein Yield

**Figure 3:**
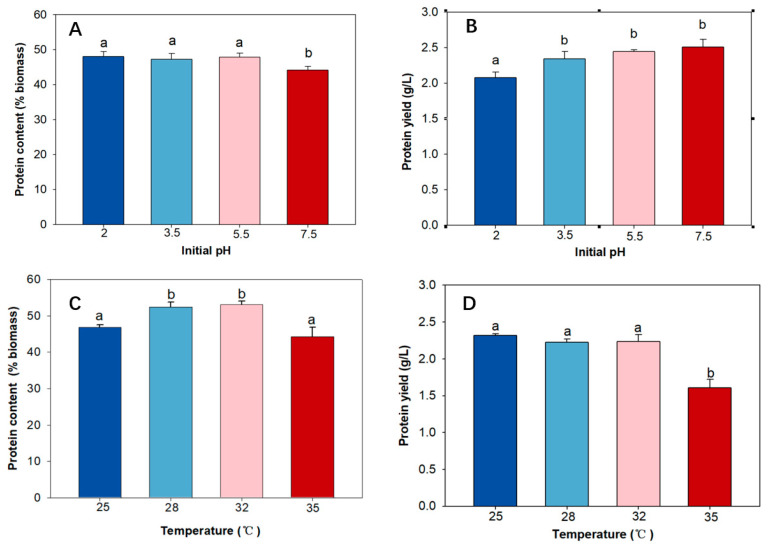
(**A**) Protein content and (**B**) protein yield of *E. gracilis* cultured with ammonium sulfate as nitrogen source under 25 °C/C/N = 17 and different initial pH. (**C**) Protein content and (**D**) protein yield of *E. gracilis* cultured with ammonium sulfate as nitrogen source/ initial pH 3.5/C/N = 17 and under different temperature conditions. Bars followed by the same letter are not significantly different (*p* < 0.05).

In the present study, *E. gracilis* was found to grow well under an initial pH of 3.5–7.5 (Figure S3). However, it had poor growth under initial pH 9.5 and partial growth inhibition under initial pH 2 (Figure S3). The protein content remained consistent with the initial pH 2–5.5, which was higher than that of the initial pH 7.5 group (44.19 ± 1.03%) (Figure 3A). This finding was partly supported by a previous study on mixotrophic cultivated *E. gracilis*, which reported that an acidic condition was more suitable for protein accumulation, displaying the highest protein content at an initial pH of 3.5 with a gradual protein decreasing trend from initial pH 3.5 to initial pH 8.5 [30]. In addition, the initial pH 2 group showed a lower protein yield (2.08 ± 0.08 g/L) than initial pH 3.5 (2.34 ± 0.11 g/L) and initial pH 5.5 (2.45 ± 0.02 g/L) (Figure 3B), suggesting that initial pH 3.5 and 5.5 were suitable initial pH conditions for protein production. However, compared to the initial pH of 5.5, the initial pH of 3.5 had an advantage in terms of anti-contamination. Therefore, the optimal initial pH for *E. gracilis* protein production was 3.5.

### 2.4. The Effect of Temperature on Protein Content and Protein Yield

The optimal growth temperature for most microalgae species is normally within the range of 15–30 °C [31]. For *E. gracilis*, the optimal temperature for growth was found to be 29 °C [32] and 30 °C [33]. As shown in Figure 3C, the protein content of *E. gracilis* displayed a non-significant difference between 28 °C (52.33 ± 1.41%) and 32 °C (53.05 ± 0.89%), which were higher than 25 °C (46.83 ± 0.70%) and 35 °C (44.29 ± 2.56%). In addition, the protein yield of *E. gracilis* cultured at 25 °C (2.32 ± 0.02 g/L), 28 °C (2.23 ± 0.04 g/L), and 32 °C (2.24 ± 0.10 g/L) was significantly higher than that cultured at 35 °C (1.61 ± 0.11 g/L) (Figure 3D), implying that *E. gracilis* was incapable of protein accumulation at 35 °C. *E. gracilis* showed the highest specific growth rate at 32 °C (Table S1). Consequently, it can be concluded that 32 °C was the most suitable temperature for protein production, as it resulted in a high protein yield and maximum specific growth rate.

### 2.5. Effect of C/N Ratio on Protein Content, Protein Yield, and Amino Acid Profile

**Figure 4:**
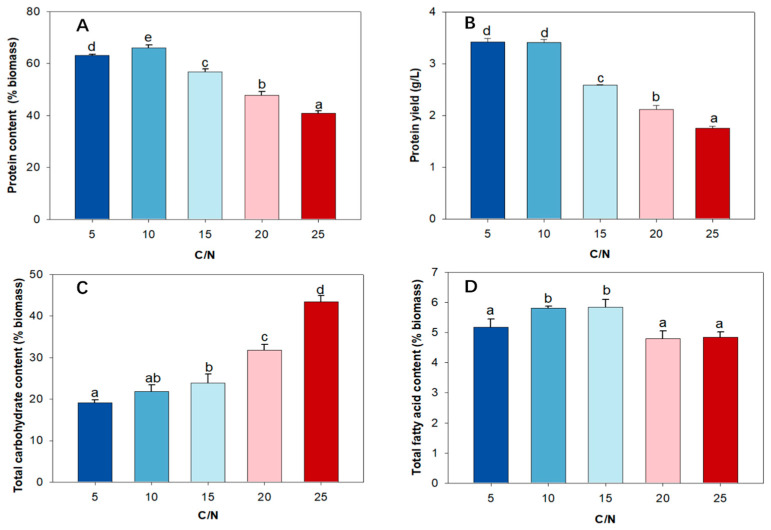
(**A**) Protein content, (**B**) protein yield, (**C**) total carbohydrate content, and (**D**) total fatty acid content of *E. gracilis* cultured with different C/N ratio under ammonium sulfate as nitrogen source, initial pH 3.5/32 °C, heterotrophic conditions. Bars followed by the same letter are not significantly different (*p* < 0.05).

To find out the impact of the C/N ratio on *E. gracilis* and further excavate its protein accumulation potential, the influence of the C/N ratio on protein biosynthesis in *E. gracilis* was studied. Figure 4A revealed a significant positive impact of the low C/N ratio for protein biosynthesis where the protein content of the C/N = 10 group was 1.6-fold of the C/N = 25 group. At an optimal C/N ratio of 10, *E. gracilis* showed a remarkably high protein content of 66.10 ± 1.12%, which is comparable to that of traditional microalgal protein sources such as *C. vulgaris* (51–58%) and *A. maxima* (60–71%) [4]. Compared with the yeast extract group in Section 3.1 (Figure 1C), the optimal protein content of *E. gracilis* was increased by 169% under C/N = 10. This emphasizes the enormous influence of the nitrogen source and C/N ratio for *E. gracilis* protein production. This result also confirmed the great potential of heterotrophic *E. gracilis* as a dietary protein source. However, the relationship between nitrogen availability and protein content was not always positively correlated. The C/N = 5 group displays lower protein content compared with the C/N = 10 group (Figure 4A), while their protein yields were at the same level (Figure 4B). A similar result was found in *C. vulgaris,* in which the decrement of the C/N ratio from 12 to 7 displayed a slight protein content drop [25]. Additionally, the high nitrogen substrate concentration (ammonium sulfate at 4360 mg/L, C/N = 5) did not inhibit the growth of *E. gracilis* (Figure S4). The high ammonium toleration of *E. gracilis* further revealed the advantage of cultivation under an initial pH of 3.5 since the ratio of toxic NH_3_ to NH_4_^+^ decreased as the pH decreased [34]. The acidic tolerance of *E. gracilis* is crucial in achieving high protein content when using ammonium sulfate as a nitrogen source. In addition, these results also suggested the potential of *E. gracilis* for acidic industries wastewater treatment, such as pharmaceutical industries, mining sites, and ammunition industries where ammonium concentrations are typically high (5–1000 mg/L) [34].

**Figure 5:**
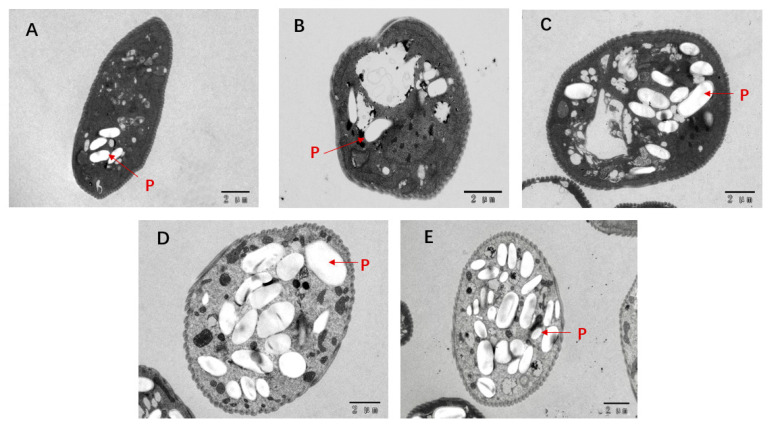
TEM images of *E. gracilis* with different C/N under heterotrophic conditions: (**A**) C/N = 5, (**B**) C/N = 10, (**C**) C/N = 15, (**D**) C/N = 20, and (**E**) C/N = 25. P represents paramylon granules in *E. gracilis* cell.

In Figure 4C,D, it could be found that the increment of the C/N ratio from 10 to 25 resulted in the carbon partitioning switch between carbohydrate (from 21.78 ± 1.61% to 43.40 ± 1.45%) and protein (from 66.10 ± 1.12% to 40.86 ± 0.93%), while the fatty acid content maintained low (<6%). This observation could also be visually confirmed by the TEM images, which showed an increase in paramylon granules in *E. gracilis* cells with an increase in the C/N ratio from 10 to 25 (Figure 5). Paramylon is a well-known storage carbohydrate of *E. gracilis* under aerobic cultivation conditions [35]. Thus, the low C/N and appropriate nitrogen source under aerobic conditions favored protein accumulation in *E. gracilis* at the expense of carbohydrates.

**Figure 6:**
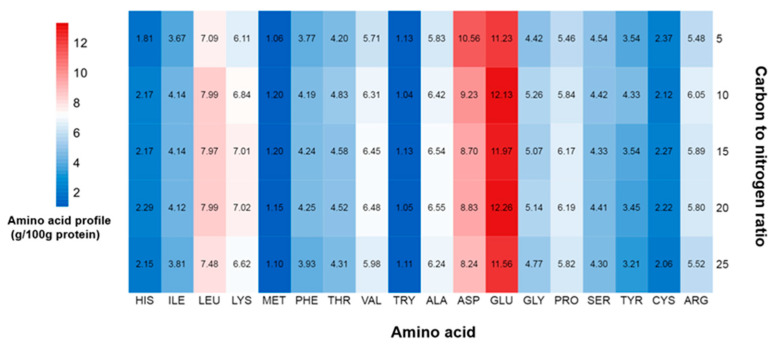
Heatmap of amino acid distribution (g/100 g protein) of *E. gracilis* cultured with different C/N under heterotrophic cultivation conditions. (HIS: histidine, ILE: isoleucine, LEU: leucine, LYS: lysine, MET: methionine, PHE: phenylalanine, THR: threonine, VAL: valine, TRY: tryptophan, ALA: alanine, ASP: aspartic acid, GLU: glutamic acid, GLY: glycine, PRO: proline, SER: serine, TYR: tyrosine, CYS: cysteine, ARG: arginine) The number showed in heatmap is the average of each amino acid content.

Considering the amino acid pattern of protein-sourced plants (e.g., soybean, oat) changed with C/N ratio [36], the amino acid profile of *E. gracilis* under different cultured C/N ratios was also analyzed. It was only found significant differences in threonine between C/N = 5 (4.20 ± 0.44 g/100 g protein) and C/N = 10 (4.83 ± 0.06 g/100 g protein) and in tyrosine between C/N = 10 (4.33 ± 0.87 g/100 g protein) and C/N = 25 (3.21 ± 0.06 g/100 g protein), which implied that the variation of C/N ratio did not significantly alter amino acid profile of *E. gracilis* (Figure 6). The stable amino acid pattern under the variation of the C/N ratio in *E. gracilis* was different from *C. sorokiniana*, which had a dramatic increase in amino acid content derived from the TCA cycle as the decrease in the C/N ratio [37].

The consistent amino acid pattern observed in different C/N ratio groups resulted in a stable *NTP*. The *k_P_* and *NTP* values were at a similar level among the five groups. The C/N = 5 group had a significantly higher *k_A_* value of 6.26 ± 0.07 than the C/N = 25 group of 6.17 ± 0.01 (Table 2). In addition, the EAAI of *E. gracilis* protein was 1.62 ± 0.05 of the C/N = 10 group, indicating superior protein quality [18]. This was comparable with *Chlorella* sp. (EAAI = 1.67) and *Spirulina* sp. (EAAI = 1.63), which are the conventional microalgal protein source [38], while it is higher than *D. salina* protein (EAAI = 1.53), which is also a novel microalgal protein source [18]. These results confirmed that the heterotrophic *E. gracilis* was a promising single-cell-protein resource with high protein content and nutritional value.

## 3. Materials and Methods

### 3.1. Microalgae Strain and Culture Conditions

*Euglena gracilis* Klebs (CCAP 1224/5Z) was purchased from the Culture Collection of Algae and Protozoa (CCAP, Oban, UK). Algal cells were maintained on an agar plate with modified Hutner’s medium at 16 °C [39]. For each experiment, a single colony of *E. gracilis* was inoculated into 20 mL modified Hutner’s medium in a 50 mL Erlenmeyer flask and cultivated in shakers (150 rpm, 25 °C) in darkness for 5 days. The algal liquid was subsequently inoculated (7.5% *v*/*v*) into a 250 mL Erlenmeyer flask with a total volume of 100 mL and shaken for 48 h in the same condition for further use as a seed.

### 3.2. Optimization of Culture Conditions

Sequential optimization experiments of the nitrogen source (yeast extract, monosodium glutamate, ammonium sulfate, sodium nitrate, and urea), temperature (25 °C, 28 °C, 32 °C, 35 °C), initial pH (2, 3.5, 5.5, 7.5), and C/N ratio (5, 10, 15, 20, 25) were conducted in 250 mL Erlenmeyer flasks with a working volume of 100 mL. The initial biomass concentration was kept around 0.4 g/L for each inoculation. The initial glucose concentration was fixed at 10 g/L (except for the sodium glutamate group to maintain the consistent C/N).

### 3.3. Determination of Biomass Concentration

Five microliters of cell culture were sampled and washed twice before being filtered on weighted GF/C film (Whatman International, Maidstone, UK) and dried in the 80 °C vacuum dryer overnight to measure the biomass concentration. The samples were collected for further analysis 12 h after they reached the stationary phase. All the sample was washed three times with deionized water and freezing dried.

### 3.4. Protein Content Analysis

The crude protein content of the sample was measured using the Dumas method [40]. Around 50 mg of the sample was wrapped in aluminum foil and combusted with high-purity oxygen gas at 900 °C. The nitrogen gas released from the sample was detected by a thermal conductivity detector (Haineng, Weifang, China). The crude protein content of *E. gracilis* was calculated with a conversion factor of 6.25 [41].

### 3.5. Photosynthetic Pigments Analysis

Chlorophyll a, chlorophyll b, and carotenoid content were determined using a spectrophotometer at the wavelength of 480, 646, and 662 nm [42]. Pigment concentration (mg/L) was calculated using the following equation:

Chlorophyll a = 11.75Ab_662_ − 2.35Ab_646_, (1)

Chlorophyll b = 18.16 Ab_646_ − 3.96 Ab_662_, (2)

Carotenoid = 4 × Ab_480_, (3)

where Ab_662_, Ab_646,_ and Ab_480_ are the sample absorbances at 662, 646, and 480 nm, respectively.

### 3.6. Amino Acid Composition Analysis

The amino acid composition analysis was proceeded by acid hydrolysis except tryptophan. Around 50 mg of lyophilized sample was acid hydrolyzed by 6 M HCl at 110 °C for 24 h under vacuum conditions. The sample was cooled down and neutralized via saturated sodium carbonate until the pH reached 7. The hydrolyzed sample (10 μL) was mixed with 70 μL of AccQ-Tag buffer and 20 μL of derived AccQ-Tag reagent solution [43]. The mixture was incubated at 50 °C for 10 min and then transferred to a micro-vial. The sample was analyzed using a Waters Alliance e2695 high-performance liquid chromatography (HPLC) system equipped with a Waters 2998 PDA detector and an AccQ-Tag amino acid column Nova-Pak C18, 4 μm (150 × 3.9 mm) (Waters, Milford, MA, USA). To analyze methionine and cysteine, samples were oxidized with performic acid before the acidic hydrolysis mentioned above.

For tryptophan analysis, 50 mg of the sample was hydrolyzed by 5 M LiOH at 110 °C for 24 h under vacuum conditions. After hydrolysis, the sample was cooled down and neutralized by 6 M HCl until the pH reached 7. The neutralized sample was filtered via filter paper and analyzed using a Waters ACQUITY ultra-performance liquid chromatography–mass spectrometry (UPLC-MS) equipped with a PDA detector and BEH C18 column (1.7 μm, 2.1 × 50 mm) [44].

The NTP was calculated according to the equation below [29].

*k_A_* = *Ei*/*Di*, (4)

*k_P_* = Σ*Ei*/*N%*, (5)

*>NTP* = (*k_A_* + *k_P_*)/2, (6)

where Σ*Ei*, Σ*Di*, and *N%* are the summation of anhydrous amino acid, the summation of the nitrogen content of each anhydrous amino acid, and the nitrogen content of *E. gracilis* sample determined by the Dumas method using the conversion factor of 6.25, respectively [29,45]. *k_A_* and *k_P_* are the upper bound and lower bound of the conversion factor, respectively [29]. *NTP*, the average value of *k_A_* and *k_P_*, is considered the best conversion factor for practical use [45].

The EAAI was calculated according to the equation [18] as follows:

EAAI = (aa_1_/AA_1_ × aa_2_/AA_2_ × ...... × aa_n_/AA_n_)^1/n^ (7)

where aa_n_ was the essential amino acid (EAA) content over total protein in the sample (mg/g protein) and AA_n_ was adult EAA content (mg/g protein) requirements issued by WHO [46]. The quality of protein was classified as superior (EAAI ≥ 1), high (1 > EAAI > 0.95), good (0.86 < EAAI ≤ 0.95), useful (0.75 < EAAI ≤ 0.86), or inadequate (EAAI ≤ 0.75) [18].

### 3.7. Total Fatty Acid Content Analysis

Twenty micrograms of lyophilized samples were methyl esterized with 1% (*v*/*v*) sulfuric acid in methanol and methylbenzene with heptadecanoic acid (C17:0, Sigma-Aldrich, St. Louis, MO, USA) as the internal standard. Samples were incubated in a 50 °C water bath overnight and analyzed using a gas chromatography–mass spectrometry (GC-MS) equipped with a DB-WAX column (30 m × 0.25 mm × 0.25 μm) (Agilent, Santa Clara, CA, USA) [47].

### 3.8. Total Carbohydrate Content Analysis

Ten micrograms of lyophilized samples were hydrolyzed by 2 mL of 12 M H_2_SO_4_ for 1 h at 35 °C. Samples were then added to 10 mL of deionized water for further hydrolyzation at 98 °C for 1 h. The hydrolyzed samples were further determined using the phenol–-sulfuric acid method [48].

### 3.9. Transmission Electron Microscopy (TEM)

The freshly prepared samples were washed with PBS buffer twice and fixed overnight with 2.5% glutaraldehyde buffer at 4 °C. The samples were washed with 0.1 M PBS buffer (pH 7.0) followed by 1–2 h fixing with 1% osmic acid. The osmic acid was then replaced by 0.1 M PBS buffer (pH 7.0) and gradient dehydrated by a graded series of alcohol. After being replaced with acetone, the samples were embedded with epoxy resin and ultrathin sectioned to 70–90 nm thickness using an ultramicrotome EM UC7 (Leica Biosystems, Solms, Germany). The ultrathin-sectioned samples were stained with acetic acid glaze and lead citrate and observed using TEM JEM1200EX (JEOL Ltd., Tokyo, Japan) operated at 120 kV.

### 3.10. Statistics Analysis

All experiments were conducted in triplicates. The results were expressed as mean ± standard deviation (SD). The statistical significance of the results was validated using a one-way analysis of variance (ANOVA) in the SPSS 26.0 software (IBM, Armonk, NY, USA); the significance level was set at *p* < 0.05.

## 4. Conclusions

In the present study, we demonstrated that ammonium sulfate could significantly enhance the protein biosynthesis ability of *E. gracilis* growing heterotrophically. The low cultural C/N ratio condition was favorable for protein production, and the protein content could reach up to 66.10% (*w*/*w*). In addition, the protein quality evaluation of the heterotrophic *E. gracilis* protein showed a high *NTP* factor (5.63) and high EAAI (1.62), further indicating its high nutritional value. These findings suggested that heterotrophic cultured *E. gracilis* has great economic potential as an alternative protein source due to its high protein content and nutritional value, acceptable color, cell wall-free nature, and dietary-approved status.

## Figures and Tables

**Table 1:** Amino acid profile (g/100 g sample) of *E. gracilis* with different nitrogen sources under heterotrophic cultivation conditions and corresponding nitrogen-to-protein conversion factor (NTP). (MSG: monosodium glutamate, EAA: essential amino acid, NEAA: non-essential amino acid, AA: amino acid, E/T: ratio of total essential amino acid content to total amino acid content).

	Yeast Extract	Ammonium Sulfate	MSG
Sum of EAA (g/100 g sample)	9.79 ± 0.50 ^a^	19.90 ± 0.80 ^c^	11.54 ± 0.55 ^b^
Sum of NEAA (g/100 g sample)	13.74 ± 0.74 ^a^	27.64 ± 1.28 ^c^	19.63± 0.89 ^b^
Sum AA (g/100 g sample)	23.53 ± 1.24 ^a^	47.53 ± 2.08 ^c^	31.16± 1.38 ^b^
E/T (%)	41.61 ± 0.13 ^a^	41.87 ± 0.21 ^a^	37.02 ± 0.59 ^b^
*k_A_*	6.16 ± 0.01 ^b^	6.17 ± 0.00 ^b^	6.11 ± 0.01 ^a^
*k_P_*	5.14 ± 0.34 ^a^	4.87 ± 0.13 ^a^	4.62 ± 0.13 ^a^
*NTP*	5.65 ± 0.17 ^b^	5.52 ± 0.06 ^ab^	5.37 ± 0.06 ^a^

^a–c^ Row values with the same superscript letter display not significantly different (*p* < 0.05).

**Table 2:** NTP and EAAI of *E. gracilis* with different C/N under heterotrophic cultivation conditions.

	C/N = 5	C/N = 10	C/N = 15	C/N = 20	C/N = 25
*k_P_*	4.71 ± 0.31^a^	5.07 ± 0.08 ^a^	5.00 ± 0.10 ^a^	5.02 ± 0.53 ^a^	4.72 ± 0.20 ^a^
*k_A_*	6.26 ± 0.07 ^b^	6.20 ± 0.05 ^ab^	6.18 ± 0.01 ^ab^	6.18 ± 0.01 ^ab^	6.17 ± 0.01 ^a^
*NTP*	5.49 ± 0.13 ^a^	5.63 ± 0.02 ^a^	5.59 ± 0.04 ^a^	5.60 ± 0.26 ^a^	5.45 ± 0.10 ^a^
EAAI	1.49 ± 0.14 ^ab^	1.62 ± 0.05 ^ab^	1.63 ± 0.03 ^b^	1.62 ± 0.17 ^ab^	1.39 ± 0.05 ^a^

^a,b^ Row values with the same superscript letter display not significantly different (*p* < 0.05).

## Data Availability

Data are contained within the article and Supplemental Materials.

## Supplementary Materials

The following supporting information can be downloaded at https://www.mdpi.com/article/10.3390/md21100519/s1. Figure S1. Carotenoid, chlorophyll a and b content of *E. gracilis* under mixotrophic and heterotrophic cultivation. Figure S2. Nitrogen assimilation pathway (GS: glutamine synthase; GOGAT: glutamate synthase; GDH: glutamate dehydrogenase; Gln: Glutamine; Glu: glutamic acid; 2-OG: 2-oxoglutarate; MSG: monosodium glutamate; AMT: ammonium transporter); Figure S3. Growth curve of *E. gracilis* cultured under different initial pH; Figure S4. Growth curve of *E. gracilis* cultured under different C/N; Table S1. The maximum specific growth rate of heterotrophic *E. gracilis* under different temperatures.
