# Supplementary material for: Optimization of Heterotrophic Culture Conditions for the Microalgae Euglena gracilis to Produce Proteins
Source: Mar Drugs. 2023 Sep 29;21(10):519. doi: 10.3390/md21100519 (PMC10608195; doi:10.3390/md21100519)
Supplement: Supplementary file 1 [file marinedrugs-21-00519-s001.zip › marinedrugs-2613229-supplementary.pdf]

Supplementary Material

Optimization of heterotrophic culture conditions for the microalgae *Euglena gracilis* to produce proteins

Weiying Xie [1,2,3,4], Xiaojie Li [2,3], Huo Xu [1,4], Feng Chen [2,3], Ka-Wing Cheng [2,3], Hongbin Liu [4,5], Bin Liu [2,3**]**

[1] SZU-HKUST Joint Ph.D. Program in Marine Environmental Science, Shenzhen University, Shenzhen, China

[2] Shenzhen Key Laboratory of Marine Microbiome Engineering, Institute for Advanced Study, Shenzhen University, Shenzhen, China

[3] Institute for Innovative Development of Food Industry, Shenzhen University, Shenzhen, China

[4] Department of Ocean Science, The Hong Kong University of Science and Technology, Hong Kong, SAR, China

[5] Hong Kong Branch of Southern Marine Science & Engineering Guangdong Laboratory, The Hong Kong University of Science and Technology, Hong Kong, SAR, China

Correspondence:

Bin Liu: liubin@szu.edu.cn

Hongbin Liu: liuhb@ust.hk

1 Supplementary Figures and Tables

1.1 Supplementary Figures

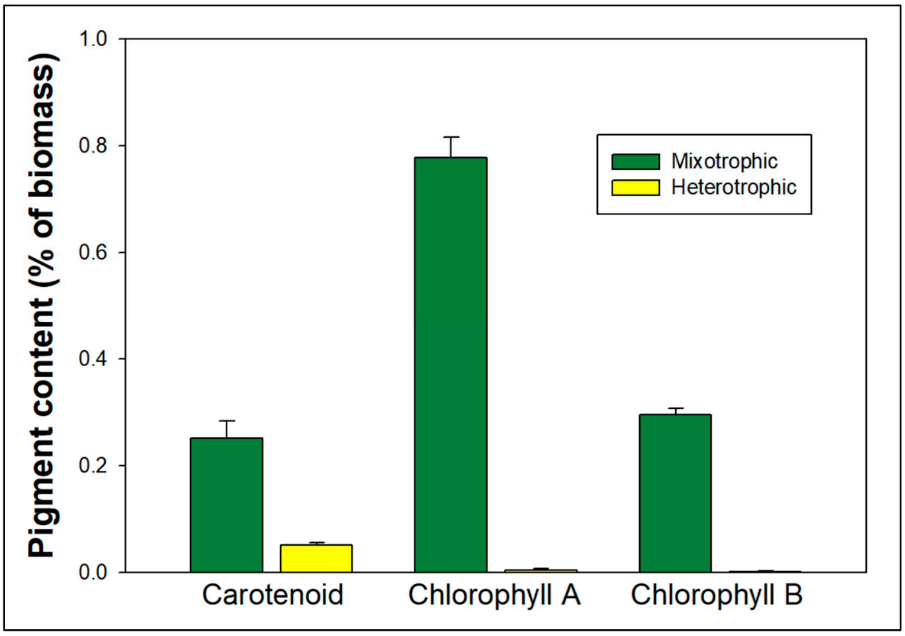

Figure S1. Carotenoid, chlorophyll a and b content of *E. gracilis* under mixotrophic and heterotrophic cultivation.

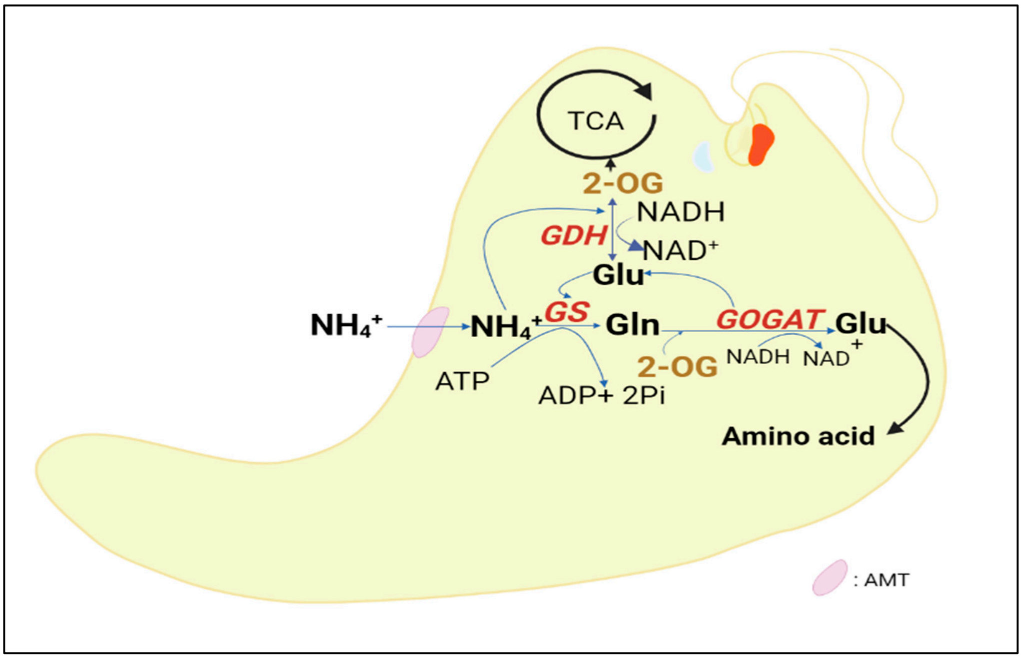

Figure S2. Nitrogen assimilation pathway (GS: glutamine synthase; GOGAT: glutamate synthase; GDH: glutamate dehydrogenase; Gln: Glutamine; Glu: glutamic acid; 2-OG: 2-oxoglutarate; MSG: monosodium glutamate; AMT: ammonium transporter).

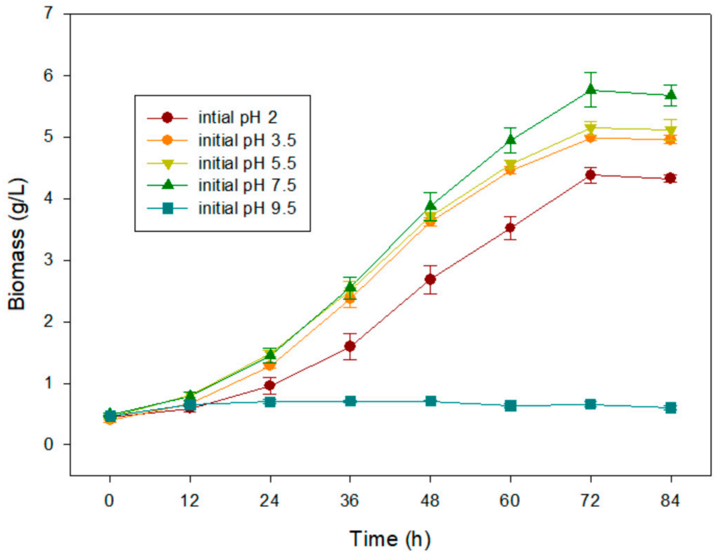

Figure S3. Growth curve of *E. gracilis* cultured under different initial pH.

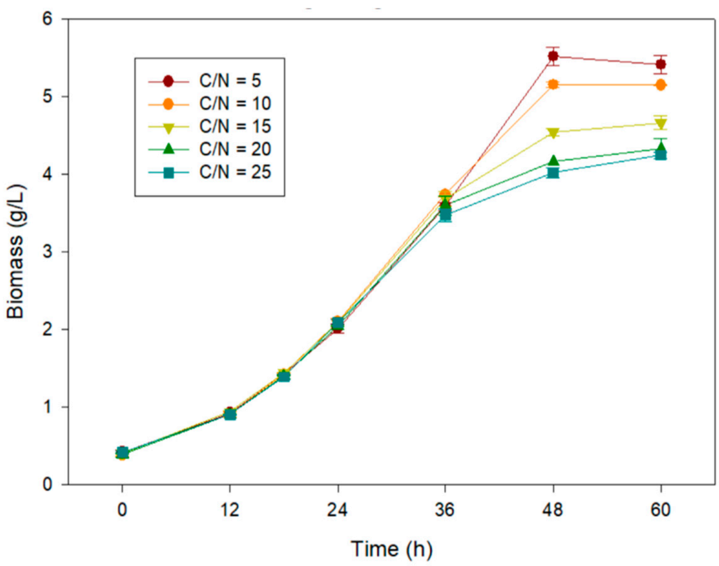

Figure S4. Growth curve of *E. gracilis* cultured under different C/N.

1.2 Supplementary Tables

Table S1. The maximum specific growth rate of heterotrophic *E. gracilis* under different temperature

Temperature (℃)	25	28	32	35
Maximum specific growth rate (/h)	0.049 ± 0.001 [a]	0.058 ± 0.001 [b]	0.069±0.002 [c]	0.061 ± 0.003 [b]

Note: [a-b] Row values with same superscript letter displays not significantly different ($p < 0.05$).
